# Nanobodies in cell-mediated immunotherapy: On the road to fight cancer

**DOI:** 10.3389/fimmu.2023.1012841

**Published:** 2023-01-25

**Authors:** Amirhosein Maali, Monireh Gholizadeh, Saba Feghhi-Najafabadi, Ahmad Noei, Seyedeh Sheila Seyed-Motahari, Shafieeh Mansoori, Zahra Sharifzadeh

**Affiliations:** ^1^ Department of Immunology, Pasteur Institute of Iran, Tehran, Iran; ^2^ Department of Medical Biotechnology, Faculty of Allied Medicine, Qazvin University of Medical Sciences, Qazvin, Iran; ^3^ Department of Medical Biotechnology, Faculty of Advanced Medical Sciences, Tabriz University of Medical Sciences, Tabriz, Iran; ^4^ Department of Biology, Science and Research Branch, Islamic Azad University, Tehran, Iran

**Keywords:** nanobodies, single domain antibodies, cancer immunotherapy, immune cell therapy, CAR, BiTE, BiKE, immune checkpoint

## Abstract

The immune system is essential in recognizing and eliminating tumor cells. The unique characteristics of the tumor microenvironment (TME), such as heterogeneity, reduced blood flow, hypoxia, and acidity, can reduce the efficacy of cell-mediated immunity. The primary goal of cancer immunotherapy is to modify the immune cells or the TME to enable the immune system to eliminate malignancies successfully. Nanobodies, known as single-domain antibodies, are light chain-free antibody fragments produced from Camelidae antibodies. The unique properties of nanobodies, including high stability, reduced immunogenicity, enhanced infiltration into the TME of solid tumors and facile genetic engineering have led to their promising application in cell-mediated immunotherapy. They can promote the cancer therapy either directly by bridging between tumor cells and immune cells and by targeting cancer cells using immune cell-bound nanobodies or indirectly by blocking the inhibitory ligands/receptors. The T-cell activation can be engaged through anti-CD3 and anti-4-1BB nanobodies in the bispecific (bispecific T-cell engagers (BiTEs)) and trispecific (trispecific T-cell engager (TriTEs)) manners. Also, nanobodies can be used as natural killer (NK) cell engagers (BiKEs, TriKEs, and TetraKEs) to create an immune synapse between the tumor and NK cells. Nanobodies can redirect immune cells to attack tumor cells through a chimeric antigen receptor (CAR) incorporating a nanobody against the target antigen. Various cancer antigens have been targeted by nanobody-based CAR-T and CAR-NK cells for treating both hematological and solid malignancies. They can also cause the continuation of immune surveillance against tumor cells by stopping inappropriate inhibition of immune checkpoints. Other roles of nanobodies in cell-mediated cancer immunotherapy include reprogramming macrophages to reduce metastasis and angiogenesis, as well as preventing the severe side effects occurring in cell-mediated immunotherapy. Here, we highlight the critical functions of various immune cells, including T cells, NK cells, and macrophages in the TME, and discuss newly developed immunotherapy methods based on the targeted manipulation of immune cells and TME with nanobodies.

## 1 Introduction

For the past 20 years, cancer incidence and mortality have been increasing, making it the leading cause of death worldwide and a huge public health concern (1). Surgery, radiation, and chemotherapy, the standard cancer treatments, have a hard time eliminating cancer cells. Cell therapy, targeted therapy, and gene therapy are other cancer treatment options (2, 3). The introduction of autologous or allogeneic cellular material into a patient for therapeutic reasons is called cell therapy. The cell-based immunotherapy harness the potential of immune cells to selectively kill cancer cells (4).

In cancer immunotherapy, the host immune system is essential in the recognition and targeting of tumor cells. The unique characteristics of the tumor microenvironment (TME), such as heterogeneity, reduced blood flow, hypoxia, and acidity, can all affect how responsive the tumor cells are to treatment (5). Although the TME's makeup varies depending on the type of tumor, common components include immune cells, stromal cells, blood vessels, and extracellular matrix. The TME is not merely a silent spectator but rather an active supporter of cancer growth (6). The cancer-associated fibroblasts, mesenchymal stem cells, and cancer-associated adipocytes are the main stromal cells in the TME. They contribute to cancer angiogenesis, invasion, and metastasis mainly through the secretion of several growth factors (such as TGF-β, EGF, and VEGF, matrix metalloproteinases, and some cytokines (such as TNF-α, IL-1, and IL-6) (7). The primary goal of immunotherapy is to modify the immune cells or the TME to enable the immune system to eliminate malignancies successfully.

**Figure 1:**
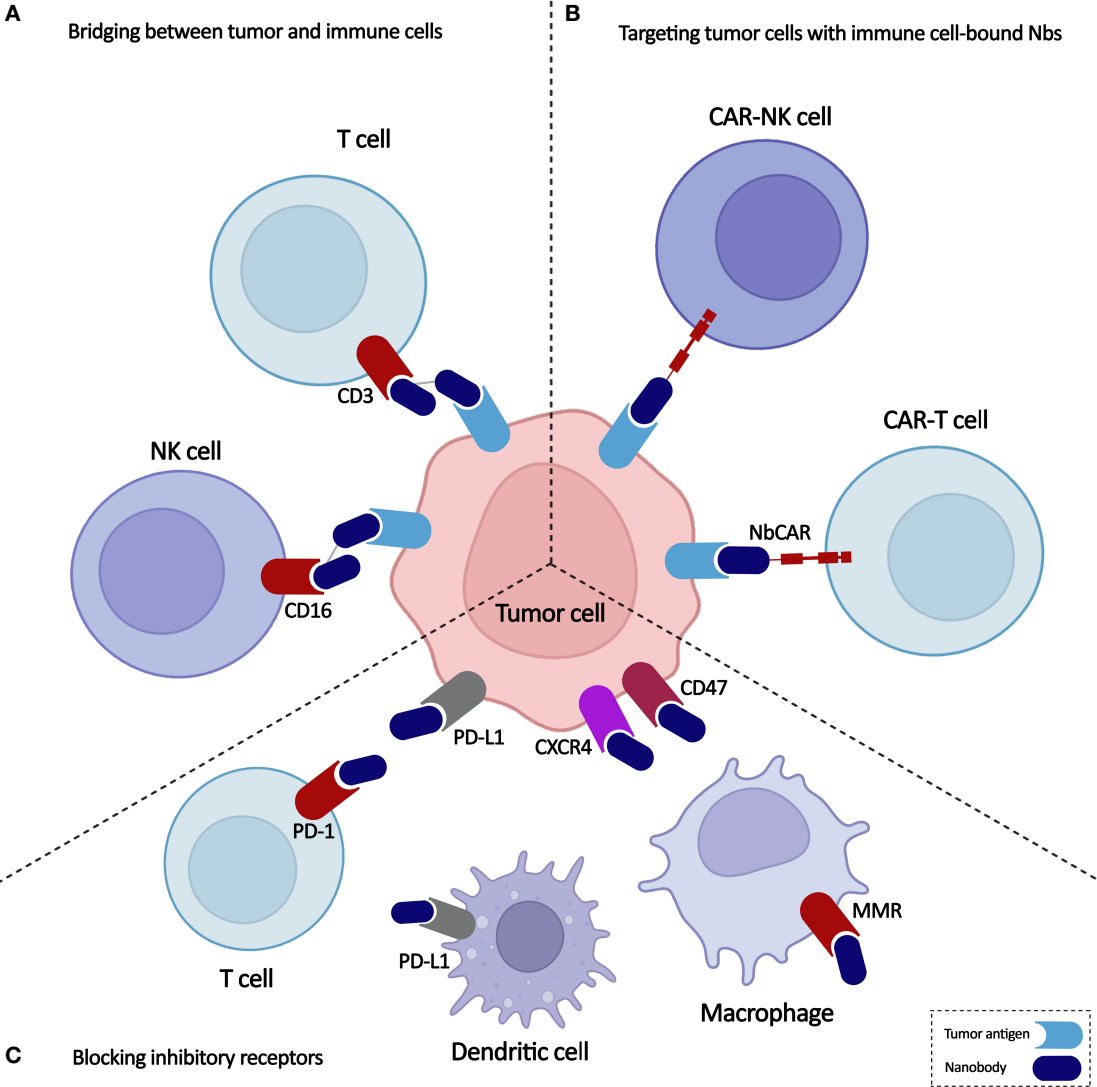
Overview on application of nanobodies in cell-based immunotherapy. Nanobodies participate in anti-cancer immune cell-mediated therapies through **(A)** bridging between tumor and immune cells, **(B)** targeting tumor cells with immune cell-bound nanobodies, and **(C)** blocking inhibitory receptors.

Monoclonal antibodies (mAbs) can bind to the activatory or inhibitory receptors expressed on immune cells and trigger changes in them that lead to the activation of the immune cells. Moreover, mAbs against tumor antigens are used to redirect the specificity of immune cells toward the malignant cells. Due to their low tumor penetrability, high manufacturing costs, and potential for developing treatment resistance, mAbs still have substantial limitations (8). Nanobodies, known as single-domain antibodies, are light chain-free antibody fragments produced from Camelidae antibodies that can be good substitutes for mAbs (9). They can promote immune cell-mediated immunotherapy either directly by bridging between tumor cells and immune cells and by targeting cancer cells using immune cell-bound nanobodies or indirectly by blocking the inhibitory ligands/receptors (**Figure 1**). Here, we highlight the critical functions of various immune cells, including T cells, NK cells, and macrophages, in the TME and discuss newly developed immunotherapy methods based on the targeted manipulation of immune cells and TME with nanobodies.

## 2 Nanobodies: The smaller variant of antibodies

**Figure 2:**
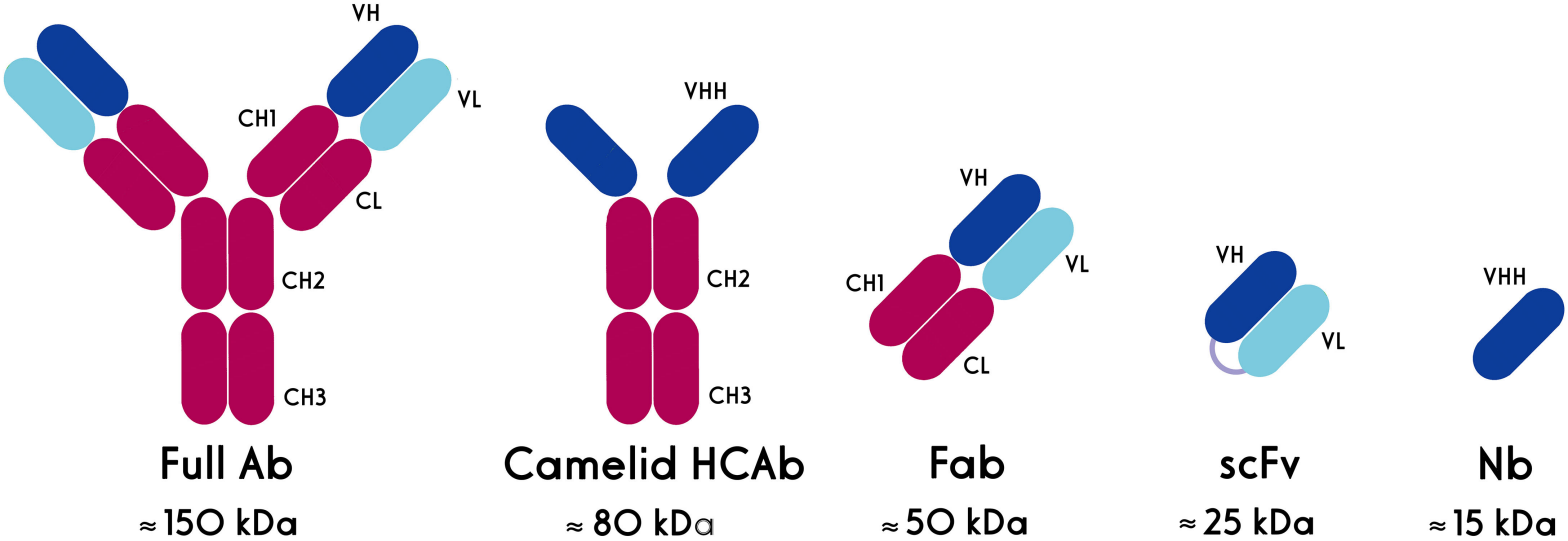
Schematic structures of conventional and heavy chain antibodies and their derivatives. HCAb: heavy chain antibody, Fab: antigen-binding fragment, scFv: single chain variable fragment, Nb, nanobody.

It has been more than three decades since the first therapeutic antibodies, which consisted of murine-derived mAbs, were approved by the U.S. Food and Drug Administration (FDA). The major disadvantages of mAbs include their immunogenicity (especially murine-derived ones) and large size. Alternatively, antibody fragments such as the antigen-binding fragment (Fab) and single-chain variable fragment (scFv) could be used in different applications (**Figure 2**). However, the short serum half-life of these fragments and their aggregation-induced immunogenicity limit their utility as both diagnostic reagents and therapeutics (8). Actually, the hydrophobic interaction of VH and VL domains limits the stability and solubility of engineered antibodies and usually leads to aggregation or mispairing of variable domains. These features show that new antibody formats are needed with the same binding specificity of antibodies but with better stability and *in vivo* characteristics (10).

Single-domain antibodies (sdAbs), also known as nanobodies or VHHs (Variable domain of Heavy chain from Heavy-chain only antibodies (HCAbs)), are derived from camelid heavy-chain antibodies. Nanobodies, as the smallest natural antigen binding domains, have dimensions in the nanometer range (~2.5 nm in diameter and ~4 nm in height) and a molecular weight of about 15 kD (8). The high-affinity nanobodies against different targets, including tumor markers, could be selected from the phage-displayed libraries through the biopanning process. They are highly soluble and do not tend to associate with other hydrophobic protein surfaces. Nanobodies have a high degree of sequence identity with human type 3 VH domains (VH3) germline sequences (11, 12), a unique property that is considered to contribute to their low immunogenicity. This reduced immunogenicity allows the prolonged and repeated administration of nanobodies in patients (13).

They can be produced easily in microorganisms, mammalian cells, or plants. Nanobody expression yield is high, whether in the periplasm of *Escherichia coli* or the cytoplasm of eukaryotic cells (14).

Thanks to their small size, intravenously administered nanobodies can rapidly extravasate from the blood circulation and deliver reagents to the target location. Moreover, their monomeric single-domain nature facilitates their genetic fusion to additional proteins, reporter molecules, or proteinaceous drugs (14, 15). Although the passage of antibodies and their derivatives through the blood-brain barrier (BBB) is a major challenge for treating brain diseases (16, 17), the small size of nanobodies may increase their chance of crossing the BBB either naturally or as a result of cancer-induced BBB leakage (18, 19). However, some nanobodies have limited BBB permeability which may be improved by different delivery methods (20), such as adeno-associated virus (AAV)-based delivery (16) and carrier-mediated transport (21).

Nanobodies have been used in different applications, including biosensing, affinity-capturing of proteins, and protein crystallization. They have been especially used for cancer therapeutics by targeting surface receptors of tumor cells such as HER2 (22), CAIX (23), TAG-72 (24), DR5 (25), c-Met (26), EGFR (27), mesothelin (28), AgSK1 (29) and CD33 (30). The main mechanisms of action of these nanobodies include suppression of downstream growth signaling and promotion of apoptosis in cancer cells (31, 32). Moreover, soluble ligands secreted by tumor cells have been targeted by specific nanobodies (9). Nanobody-based targeting of tumor ligands, including EGF (33), HGF (34), and VEGF (35), has resulted in efficient inhibition of tumor growth and metastasis.

Now, about 16 therapeutic nanobodies have entered clinical trials for various disease types (36). In 2019, Caplacizumab, a bivalent nanobody targeting von Willebrand, received approval from the FDA for the treatment of patients with thrombotic thrombocytopenic purpura (37).

## 3 Nanobody-based T-cell immunotherapy

**Figure 3:**
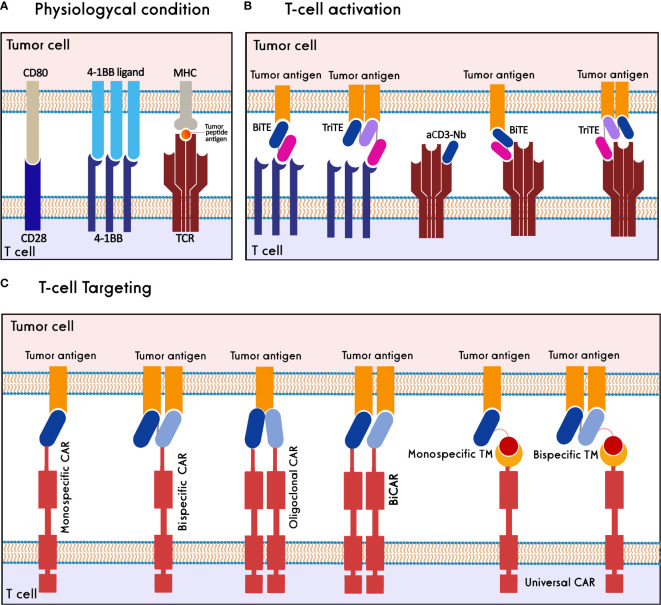
Nanobody-based T cell immunotherapy. **(A)** T cells are activated through synapsing CD3-TCR/MHC/tumor peptide antigen, CD28/CD80 and 4-1BB/4-1BB ligand. **(B)** T cells are activated against tumor cells using 4-1BB/CD3 BiTEs, 4-1BB/CD3 TriTEs, and anti-CD3-Nanobody (aCD3-Nb). **(C)** T cells target tumor antigens through monospecific CAR, bispecific CAR, oligoclonal CAR, biCAR, and monospecific-/bispecific-universal CAR-T cells.

T cells are critical components of the immune system that can be activated against cancer cells to have a functional response. The immunosuppressive cells in the TME, as well as the expression of inhibitory receptors, render T cells dysfunctional in cancer (38). Moreover, the decrease in the immunogenicity of the tumor cells, through the reduced expression of immunogenic cancer antigens or the paucity of major histocompatibility complex (MHC) class I molecules, causes the cancer cells to escape from the T cells (39). Antibodies and nanobodies could be used to activate T cells and retarget them against cancer cells (**Figure 3**).

### 3.1 Nanobody-based T-cell activation

In physiological conditions, stimulation of activatory molecules, i.e., CD3, CD28, and 4-1BB, activates T cells resulting in their proliferation and effector functions (40–42). Various studies show that T-cell activation can be induced through antibodies or antibody fragments specific to these activatory molecules. Heretofore, a CD19(scFv)/CD3(scFv) bispecific T-cell engager (BiTE), blinatumomab, has been approved for clinical administration in refractory/relapsed B- acute lymphoblastic leukemia (B-ALL) and non-Hodgkin lymphoma (NHL) (43, 44).

#### 3.1.1 CD3-based T-cell activators

The anti-CD3 nanobodies and nanobody-based CD3-targeting BiTEs have been studied as T-cell activators due to the molecular benefits of nanobodies compared to other modules. A study showed that an anti-CD3 nanobody could successfully activate T cells, raise the secretion of IL-2 and IFN-γ cytokines and suppress tumor growth in a xenograft mouse model (45). The anti-tumor property of another anti-CD3 nanobody was proved through the activation of cytotoxic T lymphocytes and inhibition of angiogenesis. In this study, angiogenic markers, i.e., VEGFR2, MMP-9, and CD31, were reduced, while activated T-cell markers such as IL-2 were increased (46).

The EgA1 anti-EGFR nanobody was used for constructing LiTE (light T-cell engager) and ATTACK (asymmetric tandem trimerbody for T-cell activation and killing cancer) bispecific engagers by combining one and three EGFR-binding nanobodies with a single CD3-binding scFv, respectively. The tetravalent BiTE, ATTACK, enhanced the binding capacity towards EGFR-expressing cells and exhibited potent target cell lysis compared to monovalent LiTE (47). Two anti-EGFR LiTEs, produced in two orientations, i.e., as EGFR(Nb)/CD3(scFv) and CD3(scFv)/EGFR(Nb), showed enhanced T-cell activation and significant inhibition of EGFR-expressing tumor cells (48). To extend the half-life of LiTE, it was fused to the human albumin sequence, which resulted in greater tumor growth suppression and a longer half-life of albumin fusion LiTE compared to the LiTE molecule without fusion (49). The anti-tumor effects of PD-L1(scFv)/CD3(scFv) and PD-L1(Nb)/CD3(scFv) BiTEs were investigated in armed oncolytic herpesvirus-1. The Nb-harboring BiTE creates a cross-linked pseudo-synapse between PD-L1 and CD3 for simultaneous immune checkpoint blockage and T-cell activation (50). Aiming to increase the avidity of antibody fragments in T-cell engagers, MART-1_27L_-HLA-A2(Nb)/CD3(scFv) BiTE was accumulated using human cartilage oligomeric matrix protein (COMP48) to form a multimeric module termed ‘combody’. This strategy led to raising the combody affinity by 10^5^-fold compared to that of the monovalent BiTE with no effect on antibody specificity (51). Moreover, for improving BiTE efficacy, a HER2-specific T-cell engager was developed by fusing two anti-HER2 nanobodies targeting non-overlapping epitopes of HER2 to an anti-CD3 Fab. This nanobody-based BiTE demonstrated potent inhibition of tumor cells compared to trastuzumab (52). A HER2(Nb)/CD3(scFv) BiTE, termed BiHC (bispecific HER2-CD3 antibody), could significantly activate T cells and increase the cytotoxicity of HER2^+^ tumor cells (53). Another BiTE against carcinoembryonic antigen (CEA), CEA(Nb)/CD3(Fab), showed potent T-cell activation in the xenograft model. The anti-tumor efficacy of this BiTE was increased *via* site-specific PEGylation (54). A CD3(scFv)/EGFR(Nb)/EpCAM(Nb) trispecific T-cell engager (TriTE) was developed to target colorectal cancer cells that could activate T cells and lyse colorectal cancer cells (55).

#### 3.1.2 4-1BB-based T-cell activators

While anti-CD28 nanobody modules have not been extensively studied, various scFv-based BiTEs were developed for targeting 4-1BB as a co-stimulator of T-cell activation; however, they were withdrawn from the clinical trials due to their high toxicity. Switching to nanobody-based 4-1BB-agonistic BiTEs can be a promising approach to overcoming T-cell activation challenges. Recently, an agonistic nanobody targeting CRD4 of 4-1BB was fused to an anti-PD-L1 nanobody. This PD-L1(Nb)/4-1BB(Nb) BiTE significantly activates T-cells and inhibits tumor cell proliferation *in vitro* and *in vivo*. In a xenograft mouse model, the nanobody-based BiTE showed reduced toxicity compared to the scFv-based one (56). A trimeric CEA(Nb)/4-1BB(scFv) BiTE was developed using the murine collagen XVIII-derived homotrimerization domain (TIE^XVIII^) which forms a hexagonal conformation (three anti-CEA nanobodies and three anti-4-1BB scFvs) (57). Also, a trimeric EGFR(Nb)/4-1BB(scFv) BiTE was constructed by this strategy (58). Both trimeric BiTEs significantly recognize 4-1BB and the corresponding tumor antigen, activate T-cells and inhibit target antigen-expressing tumor cells. Due to the multimeric formation potential of nanobody-based BiTEs and TriTEs, it is possible to evaluate co-targeting more tumor antigens and stimulatory domains, providing higher tumor specificity and lower toxicity compared to scFv and other modules.

In brief, nanobodies could provide the ability to design molecules capable of multiple binding to T cells’ activating receptors. Also, the nanobody-based T-cell activators are more efficient in infiltrating solid tumors due to their reduced molecular size. Since a HER2-targeting nanobody has entered into clinical trials (NCT04467515) and HER2(Nb)/CD3(scFv) BiTE has shown promising preclinical results, it seems that the nanobody-based anti-HER2 BiTEs have a greater chance to progress to clinical trials for treating HER2^+^ breast cancers.

### 3.2 Nanobody-based T-cell targeting

Chimeric antigen receptors (CARs) are synthetic protein molecules that redirect T cells to target tumor cells. Conventional CAR-T Cells comprise an extracellular domain of an antigen-binding scFv, a hinge domain, a transmembrane domain, an intracellular domain, and one or more costimulatory domains (59). Based on the positive characteristics of nanobodies, they were employed to establish nanobody-based CAR-T cells (NbCAR-T cells) for targeting several cancer types. So far, various strategies have been used to produce NbCAR-T cells with improved safety and efficacy, including monomeric, oligoclonal, bispecific, multispecific, and universal NbCARs. In the following, these different strategies and different tumor antigens studied in pre-clinical and clinical studies are reviewed (**Table 1**).

**Table 1:** List of nanobody-based CAR-T cells.

Functional type	Target	CAR Structure	Tumor type	Reference
Monospecific	BCMA	BCMA.Nb-CD8α-CD8αTM-4-1BB-CD3 ζ	Hematologic malignancies	(60–62)
Bi-epitopic	BCMA	BCMA.Nb-BCMA.Nb-CD8α-CD8αTM-4-1BB-CD3ζ	Hematologic malignancies	(63, 64)
Monospecific	CD38	CD38.Nb-CD8α-CD8αTM-4-1BB-CD3ζ	Hematologic malignancies	(65)
Monospecific	CD33	CD33.Nb-CD8α-CD8αTM-4-1BB-CD3ζ CD33.Nb-CD8α-CD8αTM-CD28-CD3ζ	Hematologic malignancies	(66)
Monospecific	CD20	CD20.Nb-CD8α-CD8αTM-4-1BB-CD3ζ	Hematologic malignancies	(66)
Monospecific	CD22	CD22.Nb-mutFc-CD8ATM-ICOS-4-1BB- CD3ζ	Hematologic malignancies	(67)
Monospecific	CD72	CD72.Nb-CD8α-CD8αTM-4-1BB-CD3ζ	Hematologic malignancies	(68)
Monospecific	CD7	–	Hematologic malignancies	(69, 70)
Monospecific	MUC1	MUC1.Nb-CH3CH2hinge- CD28TM-CD28-CD3 ζ MUC1.Nb-CH3CH2(hinge)_2_-CD28TM-CD28-CD3ζ MUC1.Nb-CH3CH2hinge-CD28TM-CD28-OX40-CD3 ζ MUC1.Nb-CH3CH2(hinge)_2_-CD28TM-CD28-OX40-CD3ζ	Solid tumor	(71)
Monospecific	MUC1	MUC1.Nb-IgG3Hinge-FC-CD28TM-CD28-CD3ζ	Solid tumor	(72)
Monospecific	MUC1	MUC1.Nb-IgG3-Fc&Hinge-CD28TM-CD28-CD3ζ MUC1Nb-IgG3-Fc&Hinge-Hinge-CD28TM-CD28-CD3ζ MUC1Nb-FCRIIHinge-CD28TM-CD28-CD3ζ	Solid tumor	(73)
Monospecific	MUC1	MUC1.Nb-IgG3hinge-CD28TM-CD28-CD3ζ	Solid tumor	(74)
Monospecific	PMSA	PMSA.Nb-IgG1hinge-Fc-CD28TM-CD28-CD3ζ	Solid tumor	(75, 76)
Monospecific	VEGFR2	VEGFR2.Nb-IgG1-Fc-CD28-CD28-CD3ζ	Solid tumor	(77)
Oligoclonal	TAG-72	TAG-72.Nb-IgG3-Fc&Hinge-CD28TM-CD28-CD3ζ TAG-72.Nb-IgG3-Fc&Hinge-Hinge-CD28TM-CD28-CD3ζ TAG-72.Nb-IgG3-Fc&Hinge-CD28TM-CD28-OX40-CD3ζ TAG-72.Nb-IgG3-Fc&Hinge-Hinge-CD28TM-CD28-OX40-CD3ζ	Solid tumor	(78)
Oligoclonal	HER2	HER2.Nb-IgG3-Fc&Hinge-CD28-CD28-CD3ζ HER2.Nb-IgG3-Fc&Hinge-Hinge-CD28-CD28-CD3ζ HER2.Nb-IgG3-Fc&Hinge-CD28-CD28-OX40-CD3ζ HER2.Nb-IgG3-Fc&Hinge-Hinge-CD28-CD28-OX40-CD3ζ	Solid tumor	(79)
Bispecific	CD20&HER2	CD20.Nb-IgG1-Fc-CD28TM-CD28-CD3ζ HER2.Nb-IgG1-Fc-CD28TM-CD28-CD3ζ CD20.Nb-HER2.Nb-IgG1-Fc-CD28TM-CD28-CD3ζ	Solid tumor	(80)
Bispecific	CD19&CD20	CD19.Nb-IgG4.hinge-CD8TM-4-1BB-CD3ζ CD20.Nb-IgG4.hinge-CD8TM-4-1BB-CD3ζ CD20.Nb-CD19Nb-IgG4.hinge-CD8TM-4-1BB-CD3ζ	Hematologic malignancies	(81)
Bispecific &Split	CD13&TIM3	Nb-IgG4mutant (IgG4m) hinge- CD8TM- 4-1BB, and CD3ζ CD13.Nb-CD3ζ, TIM3.scFv-CD28-4-1BB	Hematologic malignancies	(82)
Trispecific	CD19&CD20 &CD22	CD22.Nb-CD20.Nb-CD19.Nb-CD8α- 4-1BB-CD3ζ	Hematologic malignancies	(83)
Trispecific	CD33&CD123 &CLL1	TanCAR-a hinge spacer-CD8TM-41BBz--CD3ζ	Hematologic malignancies	(84)
Universal	EGFR	anti-E5B9-CD28- CD28 TM-CD28-CD3ζ	Solid tumor	(85)
Universal	EGFR	anti-E5B9-CD28-CD28 TM-CD28-CD3ζ	Solid tumor	(86)
Universal	EGFR	anti-E5B9-CD28-CD28.TM-CD28-CD3ζ	Solid tumor	(87)
Universal	CD105	CD105.Nb(C184)-CD8α.hinge-CD8α.TM-4-1BB-CD3ζ	Solid tumor	(88)

#### 3.2.1. Monospecific NbCAR-T cells

##### 3.2.1.1. Targeting hematologic malignancies by monospecific NbCAR-T cells

The most common hematologic malignancies are derived from B cells (89). Up to now, four CD19-CAR-T cell products to treat B-ALL and B-NHL and two BCMA-targeted CAR-T products to treat multiple myeloma have been approved by the FDA. The most important tumor antigens targeted by NbCAR-T cells against B cell malignancies include CD19, CD22, CD20, CD33, and CD72. Multiple myeloma, the second most common hematologic malignancy, has been targeted by NbCAR-T cells against BCMA and CD38 (65).

Three second-generation CD33-NbCARs could kill the target tumors and increase the levels of IL-2 and IFN-γ cytokines, while they had no effects on negative target tumor cells. The comparison of constructs, including 4-1BB or CD28 costimulatory domains, showed that 4-1BB-based constructs, unlike CD28-based ones, could control the new CD33^+^ THP1 tumor cells added to the medium after 7 days. Moreover, CD20-NbCAR-T cells with a 4-1BB costimulatory domain could also completely destroy subcutaneous tumors in less than 20 days (66). Three third-generation CD22-NbCAR-T cells have been individually developed using three different high-affinity nanobodies. Among them, the CD22MN-NbCAR-T cells, which harbored the nanobody with the least affinity, could effectively inhibit the tumor burden in laboratory mice. This implies that the high affinity of the targeting moiety does not correlate with strong cytotoxicity of NbCAR-T cells (67).

The CD72-NbCAR-T showed potent cytotoxic activity and strong degranulation against CD72^+^ cells and primary B-ALL samples, comparable to CD19-CAR-T cells. These CD72-specific CAR-T cells were effective even on the cells knocked out for CD19, although CD19-CAR-T activity was reduced (68). The CD7-NbCAR-T cells were able to eliminate abnormal T cells and overcome the fratricide of CAR-T cells. CD7-NbCAR-T cells need to be further investigated as a suitable therapeutic potential for the treatment of patients with T-cell malignancies. An allogeneic CD7-NbCAR-T has been designed, which avoids the expression of the CD7 cell surface to minimalize fratricide. Evaluation of its safety and efficacy proved that all side effects were both reversible and controllable except in one patient (70).

The monovalent humanized NbCAR-T cells developed against BCMA antigen showed 88.89% progression-free survival in R/R (relapsed/refractory) multiple myeloma patients (60). Unlike the scFv-CARs, these NbCARs were evenly distributed on the membrane surface of T cells. They could recognize and kill tumors with high BCMA expression more powerfully than tumors with low BCMA expression (63). A bi-epitopic NbCAR-T (LCAR-B38M) targeting two different epitopes of BCMA showed ORRs (overall response rates) and CRs (complete responses) varied from 80% to 94.8% and from 56% to 76%, respectively (62, 64). The CD38-NbCAR-T cells exposed to CD38^+^ tumors could proliferate effectively, kill the target cells, and reduce the tumor size in an *in vivo* evaluation. There were few cytotoxic effects against normal CD38^+^ cells, such as B cells, adult T cells, and NK cells (65). In spite of the low expression of CD38 on T cells (90), just a small percentage of T cells were killed by CD38-NbCAR-T. The transduced T cells were effectively proliferated and lived for a long time without functional defects (65).

##### 3.2.1.2 Targeting solid tumors by monospecific NbCAR-T cells

Unlike CAR-T cell therapy against hematological malignancies, limited clinical success has been observed in CAR-T cell therapy against solid tumors due to facing several challenges, including tumor antigen heterogeneity, trafficking and infiltration into tumor tissue, and immunosuppressive TME (88). The most important solid tumor antigens targeted by NbCAR-T cells include MUC1, prostate-specific membrane antigen (PSMA), HER2, and CD105.

Two designed MUC1-NbCAR and pFKC8 (containing human caspase 8 and two modified domains of FKBP12) constructs were co-transfected into Jurkat T cells. After the addition of dimerizer, transfected T cells were reduced by 91% (71). MUC1-NbCAR-T cells could produce IL-2 cytokine, proliferate and lyse MUC1^+^ cells (72, 74). The introduction of phiC31 integrase in MUC1-NbCAR-T cells led to efficient and stable transduction of constructs into the Jurkat cells (73). Immunohistological evaluation of the prostate samples proved that the higher the expression of PMSA, the more severe cancer (91). The PSMA-NbCAR-T cells could express the CD69 activation marker, produce the IL-2 cytokine, and inhibit PSMA^+^ tumor growth (75).

##### 3.2.1.3 Targeting tumor stroma and vasculature by monospecific NbCAR-T cells

Cancer cells often create the vascular system around the tumor to grow more rapidly. Since the expression of VEGF and VEGFR in tumors is related to their angiogenesis and metastasis (92), tumor vasculature has been a major target for CAR-T cells. The VEGFR2-NbCAR-T cells could efficiently lyse the VEGFR2^+^ tumor cells and produce IFN-γ and IL-2 cytokines (77). EIIIB, a fibronectin splice variant, is overexpressed by the tumor stroma and neovasculature, which makes it a potential target candidate for CAR-T cell therapy. EIIIB-NbCAR-T cells could delay the tumor growth, interfere with the blood supply to the tumor and enhance immune cells infiltration into the TME (93).

#### 3.2.2 Oligoclonal NbCAR-T cells

Numerous studies have shown that T cells can target tumor antigens with an oligoclonal pattern in tumor tissues, thus reducing the likelihood of antigen escape (94). Two different studies used this approach: one to design TAG72-NbCAR-T cells (78) and another one to develop HER2-NbCAR-T cells (79). When TAG72-NbCAR-expressing oligoclonal T cells were stimulated by the TAG72^+^ tumor cells, they resulted in the proliferation of CAR-T cells dependent on the target antigen and secretion of IL-2 cytokine. The designed system may avoid CAR immunogenicity and prevent the escape of the tumor cells (78). The function evaluation of the HER2-NbCAR-T cell exhibited that their cytokine secretion, proliferation, and cytotoxic activity were higher than their monoclonal counterparts (79).

#### 3.2.3. Bi- and multispecific NbCAR-T cells

One of the major drawbacks of monospecific CAR-T cells is the recurrence of the disease due to a mutation that results in the removal or reduction of the relevant tumor antigen expression. This drawback can be eliminated by designing CAR-T cells targeting more than one tumor antigen. Based on this theory, researchers have shown that the use of bispecific, tandem, and a combination of two single CAR-T cells can reduce these drawbacks (95, 96).

Three NbCAR-T cells, one tandem form (a single CAR structure consisting of two distinct antigen recognition domains targeting two tumor antigens), and two distinct monospecific forms, were designed against HER2 and CD20 tumor antigens. The efficiency of the tandem CD20-HER2-NbCAR-T cells was better than that of two monospecific NbCAR-T cells, and the efficiency of the CD20-NbCAR-T cell was the lowest. It was assumed that the distance between the CD20-Nb domain to the cell membrane of T cells was much longer than the distance between the HER2-Nb to the T cell’s membrane. Therefore, it is possible that inhibitory phosphatases enter this large immunological synapse and disrupt the activity of T cells (80). Three CAR-T constructs (CD20-NbCAR-T, CD19-NbCAR-T, and tandem CD20-CD19-NbCAR-T) were exposed to the primary cancer cells obtained from patient-derived (PD) tumor samples. These NbCAR-T cells could lyse the primary cancer cells and showed increased expression of CD69 (81). A bispecific and split CAR-T (BissCAR) was produced against CD13, and TIM3 antigens of AML cells, in which CD13-Nb was linked to CD3ζ signaling and anti-TIM3-scFv was linked to two costimulatory domains, CD28 and 4-1BB. BissCAR produced more cytokines when exposed to CD13^+^TIM3^+^ cells (mimicking leukemic stem cells) compared to CD13^+^TIM3^-^ cells (mimicking normal hematopoietic stem cells (HSCs)) because both activating and stimulatory signals were activated. BissCAR injection into NSG mice with PD AML resulted in complete tumor elimination with reduced toxicity to HSCs (82).

A trispecific NbCAR-T (triNbCAR-T), LCAR-AIO, simultaneously targets three different tumor antigens, including CD19, CD20, and CD22, to treat patients with recurrent B cell malignancies. Compared to CD19-scFvCAR-T, LCAR-AIO showed greater cytokine production and lytic activity against target cells. When LCAR-AIO was exposed to cells whose CD19 tumor antigens had been knocked out, it maintained its lytic activity, which means these cells may prevent tumor escape in CD19^-^ patients. Compared to its monospecific counterparts, LCAR-AIO showed better T-cell proliferation, longer shelf life, and superior tumor eradication efficiency in an NCG murine model (83). In another study, triNbCAR‐T cells targeting CD33, CD123, and CLL1 tumor antigens could exhibit cytolytic activity equal to or greater than their monospecific counterparts and high levels of IFN-γ and IL-2 cytokines when exposed to CD33^+^ or CD123^+^ only tumors, whereas they produced lower levels of these cytokines when exposed to CLL^+^ only tumors (84).

#### 3.2.4 Universal NbCAR-T cells

Despite of obtained remarkable successes, all of the FDA-approved CAR-T cells are being made from autologous T cells and target only one cancer antigen. These autologous CAR-T cells with a fixed antigen specificity have several negative points, involving high cost and long-lasting manufacturing, an inherent risk of product failure, and limited efficacy due to tumor antigen escape (97). Also, conventional CAR-T cell therapies may cause some side effects, such as on-target/off-tumor reactions, cytokine release syndrome (CRS), and neurotoxicity, which threaten the life of patients (98). Because these engineered CAR-T cells are inherently active, their activities and specificities are permanent and not easily controllable. Therefore, two universal systems were designed to solve these problems: i) universal CAR-T cells and ii) universal T cells (97).

##### 3.2.4.1 Universal nanobody-based CAR-T cells

These universal CAR-T cells separate the conventional CARs into two modules: i) the signaling module or uniCAR module, which harbors a binding moiety to a specific epitope combined with the intracellular signaling domains *via* a hinge and the transmembrane regions, and ii) a target module (TM), which is a bispecific fusion molecule with one binding domain directed against a tumor-associated antigen (TAA) and a part (can be an scFv, an epitope, or a small molecule) specifically recognized by the signaling module (99). To date, four diverse types of universal CAR-T cells have been developed: (i) antibody-dependent cellular cytotoxicity receptors, (ii) bispecific protein-mediated linkage, (iii) anti-tag CARs, and (iv) tag-specific interactions (100). These adaptor CAR (adCAR) platforms can switch on/off the CAR-T cells to control their activity (101). Moreover, there is the capability to simultaneously or sequentially target different TAAs (99).

The Nb-adCAR-T cells were produced based on the E5B9 peptide tag, derived from nuclear antigen La-SS-B, and anti-E5B9 scFv to split the intracellular signaling domain from the antigen-binding domain (86). These engineered T cells could target and lyse EGFR^+^ tumor cells in a TM concentration-dependent manner. Although the deletion of TM participating in the Nb-adCAR-T cell complex was delayed, free TM was deleted more rapidly (85). Because of the higher avidity of the bivalent anti-EGFR TMs, they could direct the Nb-adCAR-T cells to tumor cells with low EGFR expression levels, while the monovalent TMs could only direct the Nb-adCAR-T cells to tumor cells with high EGFR expression levels (86). In another study, the EGFR-scFv-TM was compared with the EGFR-Nb-TM. The quantities of different cytokines were similar in the presence of both Nb- and scFv-TMs. The scFv-TMs could induce the lysis of tumor cells with low EGFR expression levels more efficiently than the Nb-TMs, which may increase the risk of targeting healthy tissues (87).

##### 3.2.4.2 Universal T cells

Universal T cells are prepared by disrupting MHC loci (HLA-A) or TRAC loci of the endogenous α or β subunits at the genomic level (97). Universal CD105-NbCAR-T cells were designed by CRISPR/Cas9 method for solid tumor immunotherapy. In a human tumor xenograft model, universal CD105-NbCAR-T cells repressed the growth of CD105^+^ tumors, decreased tumor weight, and increased the lifespan of mice (88).

Since nanobodies can be easily reformatted into multi-domain structures, NbCAR-T cells are easier to engineer compared to scFv-based CARs. According to the clinical trial studies such as CD19/20 bispecific NbCAR-T cells against B cell lymphoma (NCT03881761) and BCMA-NbCAR-T cells (NCT03664661) and LCAR-B38M-NbCAR-T cells against multiple myeloma, it seems that NbCAR-T cells targeting blood cancers have the more likelihood to reach FDA approval.

## 4 Nanobody-based NK cell immunotherapy

**Figure 4:**
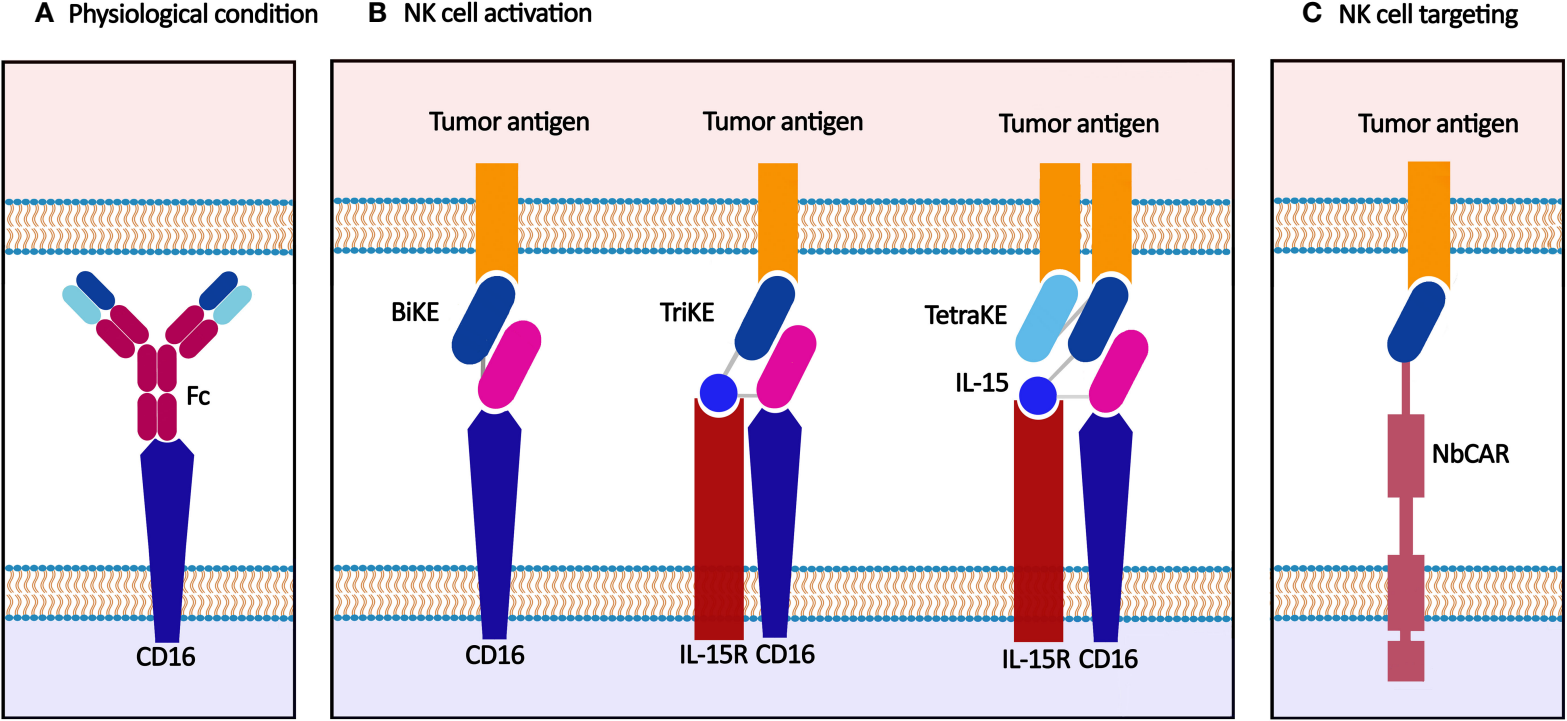
Nanobody-based NK cell immunotherapy. **(A)** NK cells are activated through synapsing Fc and CD16 in physiological conditions. **(B)** BiKE, TriKE, and TetraKE target tumor antigen(s) and IL-15R, another functional element on the NK cell membrane, e.g., CD16. **(C)** CAR-NK cell recognizes the tumor antigen and exerts anti-tumor effects.

Nanobody-mediated NK cell immunotherapy is a promising tool for non-specific tumor cell recognizing and targeting, focusing on the nature of NK cells (**Figure 4**). In physiological conditions, NK cells target tumor cells through the downregulation of HLA on tumor cells (102). They could be engineered with CARs to target different tumor antigens. Moreover, NK cells may be triggered using appropriate mAbs and antibody fragments, including nanobodies, to eliminate cancer cells.

### 4.1 Nanobody-based NK cell activators: BiKEs, TriKEs, and TetraKEs

The activation of NK cells occurs through antibody binding to CD16, a primary activator domain on NK cells. Furthermore, when CD16 is targeted, the NK cells act as cytotoxic effectors through CD16-mediated antibody-dependent cellular cytotoxicity (ADCC) (10, 103). Various soluble anti-CD16 nanobodies are established and characterized to activate NK cells again in tumor cells (in mono-, bi-, tri-, and tetraspecific manners) (104).

Bispecific and trispecific killer cell engagers (BiKEs and TriKEs) are antibody-based small molecules that create an immune synapse between the tumor and NK cells. BiKEs target a tumor antigen and another functional element on the NK cell membrane, e.g., CD16, NKG2D, and NKp46, while TriKEs target one more element (commonly a tumor antigen or IL-15).

Various preclinical studies have examined the anti-tumor potency of BiKEs and TriKEs. A CD16(Nb)/CEA(Nb) BiKE was developed for the simultaneous targeting of CD16 and CEA^+^ ovarian and colorectal cancer cells. This anti-CEA BiKE showed significant NK cell activation and suppressed cancer progression in a mouse xenograft model (105). Another anti-CEA BiKE comprising anti-CD16 and anti-CEA nanobodies could effectively recruit NK cells and showed significant *in vivo* tumor growth inhibition (106). A llama anti-CD16 nanobody was fused to three different nanobodies targeting CD19, HER2, and EGFR cancer antigens to create anti-CD16/CD19, anti-CD16/HER2, and anti-CD16/EGFR BiKEs, respectively. They induced significant target-specific activation and ADCC response in NK cells against target antigen-expressing tumor cells (107). The anti-CD16/CD30 BiKE, which targets the lymphoma antigen CD30, was enrolled in a clinical trial on relapsed/refractory Hodgkin’s lymphoma (HL) patients, which showed a high overall response rate in patients with CD30^+^ T-cell malignancies (NCT03192202). This CD30/CD16 BiKE was developed in a tetravalent manner, providing more half-life than the bivalent form (108, 109).

IL-15, physiologically secreted by activated monocytes/macrophages, activates NK cells *via* synapsing to IL-15R expressed on the NK cell membrane and stimulated NK cell expansion. CD16(Nb)/IL-15/CLEC12A(scFv) TriKE was designed for targeting acute myeloid leukemia (AML) *via* IL-15-activated NK cells. This camelied/human TriKE successfully targeted CLEC12A expressed on leukemic hematopoietic stem cells in AML patients with no bonding to normal hematopoietic stem cells (110). Moreover, CD16(Nb)/IL-15/HER2(scFv) TriKE could significantly activate the NK cells and inhibit the proliferation of HER2^+^ cancer cells *in vitro* (SKOV3 and SKBR3 cells) and *in vivo* (xenograft NGS mice models) (111).

Considering valuable properties of nanobodies, novel NK cell engagers may be developed by substituting scFv modules with existing nanobodies against different tumor antigens. For example, anti-EpCAM nanobody-based TriKEs could be developed for stimulating NK cells’ cytotoxicity in colorectal cancer using EpCAM-specific nanobodies (112, 113). Also, an anti-CD33 nanobody which was developed for targeting AML cells (30), could be used instead of anti-CD33 scFv in CD16(scFv)/IL-15/CD33(scFv) TriKE. This trivalent engager could significantly activate NK cells and induce NK-mediated ADCC against CD33 antigen expressed on AML cells and myeloid-derived suppressor cells (MDSCs) (114). Furthermore, CD16(scFv)/CD19/CD22(scFv) TriKE was developed for targeting CD19 and CD22 (leukemia/lymphoma antigens) and CD16, simultaneously (115). Since anti-CD19 and anti-CD22 nanobodies have been developed for targeting leukemia/lymphoma cells (67, 116), they can be used in nanobody-based NK cell engagers. Also, the scFv modules applied in CD16/IL-15/CD133/EpCAM TetraKE can be substituted with their nanobody counterparts for effective targeting of carcinoma antigens simultaneously (117, 118).

In conclusion, nanobodies are potent tools for activating NK cells through NK cell-activating receptors, especially CD16. The scFv-based NK cell engagers have given promising results in clinical trials targeting CD30 (NCT04101331), CD33 (NCT03214666), BCMA (NCT04434469), EGFR (NCT04259450), and HER2 (NCT04143711). Considering that nanobodies targeting HER2 (NCT04467515) and BCMA (as NbCAR-T cells) (NCT03664661) have reached clinical trials, nanobody-based NK cell engagers targeting HER2 and BCMA have more chance to be effective against HER2^+^ solid tumors and multiple myeloma, respectively.

### 4.2 Nanobody-based targeting of NK cells

CAR-NK cells target tumor antigens expressed on the surface of tumor cells and simultaneously benefit from the NK cells' cytotoxic effects. CAR-NK cells have advantages over CAR-T cells; including lower on-target/off-tumor toxicity due to their shorter lifespan than CAR-T cells, lower CRS and neurotoxicity due to their limited cytokine secretion profile, activation by their natural cytotoxicity receptors in a CAR-independent manner, killing tumor cells through CD16-mediated ADCC, and availability of allogeneic sources of NK cells, i.e., NK92 cell lines, peripheral blood mononuclear cells, umbilical cord blood, and induced pluripotent stem cells (iPSCs) (119). Nanobodies are utilized in the extracellular recognition domain of the chimeric receptors in CAR-NK cells. Compared to scFvs, nanobodies have a more efficient surface expression level and less cross-reactivity in CAR-NK cells. Also, nanobody-based CAR-NK cells have fewer recipient immunogenic responses than CAR-NKs targeted with murine-derived scFvs. Furthermore, multidomain applications of nanobodies are more retained due to the smaller size than scFv (120).

In a study, monovalent anti-CD7 nanobody (VHH6) and bivalent (VHH6-VHH6) nanobodies were fused to a CAR construct and expressed on the surface of NK-92MI cells. The results indicated the enhanced activation and cytotoxicity of CAR-NK cells that led to significant inhibition of T-ALL cells (121). In another study, an anti-CD38 nanobody was used for targeting CD38^+^ MM/Burkitt lymphoma cells by CAR-NK cells. These cells could successfully lyse the primary human multiple myeloma cells and deplete CD38^+^ myeloma cells in human bone marrow-derived samples (122).

In another strategy, TCR-like nanobodies were applied to target intracellular tumor antigens presented by the HLA molecules. In an NK cell-based anti-melanoma experiment, GPA7, a TCR-like nanobody, was fused to the intracellular domain of CD3ζ to target the melanoma-associated gp100/HLA-A2 complex expressed in melanoma cells. The engineered NK92 cells could effectively recognize melanoma cells and inhibit their growth in a mouse xenograft model (123). Nanobody conjugation to NK cells was used for the simulation of CAR therapy. NK92 cells were conjugated with an anti-EGFR nanobody through the glycoengineering approach. The conjugated NK cells could target EFGR-overexpressing tumors and exhibit potent cytotoxic activity toward them (124).

Since then, there have been multiple clinical trials on CD7- and CD38-targeting CAR-T cells to treat a number of different blood and bone marrow cancers (125). Moreover, a phase I/II trial was conducted to evaluate the safety and efficacy of anti-CD7 CAR-NK cells in CD7^+^ leukemia and lymphoma patients (NCT02742727). Based on the hopeful outcomes of treatment with CAR-T and CAR-NK cells targeting CD7 and CD38 markers, as well as the success *in vtiro* development of nanobody-based CAR-NK cells against these tumor antigens, it seems that nanobody-based CD7 and CD38 CAR-NK cells will advance to clinical stage investigations for the treatment of leukemia, lymphoma, and myeloma.

## 5 Nanobody-based targeting of macrophages

Macrophages are highly plastic immune cells that comprise phenotypically different populations in various cancers (126). They have exhibited dual functions based on the microenvironmental cues in cancer development, including preventing tumor growth in the early stages and promoting it in the later ones (127). One of the most important reasons for macrophage targeting in cancer immunotherapy is their high ability to infiltrate (comprise >50% of the tumor mass) into the TME (128). Although T cells are the main immune cells for the removal of tumor cells, they have low infiltration and are often suppressed by TME inhibitory factors (129).

Macrophages are functionally divided into two subtypes: classically activated macrophages (M1) and alternatively activated macrophages (M2). M1 macrophages have anti-tumor activity and secrete pro-inflammatory cytokines such as IL-1β, IL-6, IL-12, IL-23, TNF-α nitric oxide, and CXCL9 and CXCL0 (130, 131). LPS and T helper (Th) 1 cytokine, like IFN-γ polarize macrophages towards the M1 phenotype (132, 133). M1 cells, in turn, have a positive influence on Th1 cells and boost anti-tumor responses. Anti-inflammatory responses of macrophages are relevant to the M2 phenotype. IL-4 released by Th2 cells and the production of IL-4, IL-10, or IL-13 by tumor cells polarize macrophages into an M2 phenotype (131, 134). Tumor-associated macrophages (TAMs) localize in the TME, usually have an M2 phenotype, and comprise the most number of immune cells in TME (135). TAMs remodel the TME by releasing the inhibitory cytokines, including TGF-β and IL-10, and expressing immune checkpoint ligands to inhibit infiltration and anti-tumor activity of other immune cells, especially cytotoxic T cells. A subset of TAMs that overexpresses macrophage mannose receptors (MMR, CD206) under hypoxic conditions refers to MMR^high^MHC-II^low^ TAMs. They are strongly involved in angiogenesis and immunosuppression of the TME (136). Therefore, targeting macrophages, especially TAMs, can be a promising opportunity for boosting immunotherapy of various malignancies.

**Figure 5:**
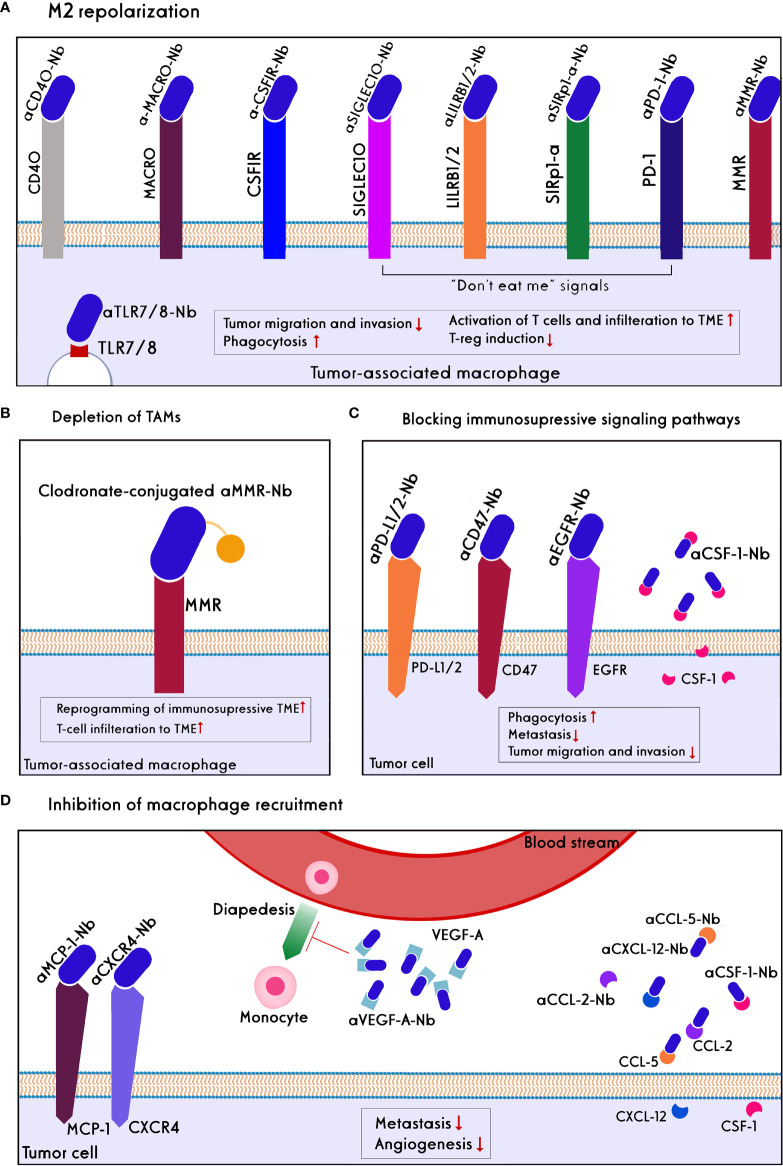
Approaches of nanobody/antibody-based targeting of Macrophages. Direct targeting strategies include: **(A)** M2 repolarization: CD40 and TLR7/8 agonists, the blockades of MACRO, CSF1R, MMR, and blocking of ‘don’t eat me’ signals such as PD1, SIRP-a, LILRB1/and 2, and SIGLEC10 on TAMs lead to repolarization of M2 to M1 phenotype in the TME. As a result, tumor migration, invasion, and Treg induction decline, whereas phagocytosis, T-cell activation, and tumor infiltration increase. **(B)** TAMs depletion using anti-MMR conjugated clodronate, as a bisphosphonate, increase reprogramming of immunosuppression of TME and T-cell infiltration. Indirect targeting strategies include: **(C)** blocking of immunosuppressive signaling pathways: inhibition of CSF-1 ligand secreted by tumor cells, blocking of don’t eat me signals like CD47 and PDL1/2 on the tumor cells, as well as blocking EGFR on the tumor cells prevent scape of the tumors from phagocytosis and decrease metastasis, tumor migration, and invasion. **(D)** Inhibition Macrophage recruitment: Chemokine of MCP-1 on the tumor cells, soluble chemoattractants such as CCL2, CLL5, CXCL12, CSF-1, VEGF-A has secreted by tumor cells and bystander cells to recall macrophages to the TME. Anti-VEGF-A nanobody/antibody reduces angiogenesis. Nanobodies/antibodies against these chemokines prevent the diapedesis of monocytes into the TME and then their differentiation to the immunosuppressive TAMs. Also, the blockade of the CXCL12/CXCL4 axis reduces metastasis.

Different macrophage-based strategies have been used to enhance cancer immunotherapy, including TAMs depletion, M2 repolarization, blocking of checkpoint immune ligands/receptors on macrophages and “don’t eat me” signaling, and preventing infiltration of macrophages into the TME (**Figure 5**).

Recently, the use of nanobodies to target macrophages have been exhibited improved therapeutic effects combined with high safety compared to mAbs in multiple malignancies. For instance, simultaneous inhibition of the CCL2 and CCL5, which are necessary chemokines for attracting TAMs to the TME and upregulated on hepatocellular carcinoma tumor cells, by a bispecific nanobody (BisCCL2/5i) demonstrated more survival benefits than the combination of two full-length antibodies or two small molecules against CCL2 and CCL5 *in vivo*. Delivery of the BisCCL2/5i mRNA encapsulated in an approved lipid nanoparticle led to not only a reduction of more than 50% of M2 macrophages and mitigation of intratumoral macrophages trafficking but also significantly polarized M2-TAMs to an M1-phenotype leading to the increased M1/M2 ratio and improved T-cell infiltration. Combination therapy using BisCCL2/5i nanobody and a trimeric PD-L1 inhibitor triggered a robust anti-tumor response and long-term survival in primary and metastatic liver malignancies (137).

The binding of CD47 on cancer cells to signal regulatory protein-a (SIRPa) on macrophages results in the escape of cancer cells from phagocytosis (138). Blockade of the CD47/SIRPa pathway can enhance macrophage-mediated phagocytosis (MMP), increase the frequency of TAMs in TME, and reduce tumor growth (139). Clinical mAbs that target CD47 have some side effects (e.g., RBC hemagglutinin, platelet aggregation) that may limit their application (140). The pre-clinical studies showed that HuNb1-IgG4, a fusion protein containing an anti-human CD47 nanobody and a human IgG4 Fc fragment, could overcome these drawbacks. This nanobody showed a low affinity for RBCs that led to less toxicity while being able to specifically recognize the human CD47 and bound to it with a higher affinity than B6H12 (an anti-CD47 mAb). HuNb1-IgG4 nanobody exhibited superior anti-tumor efficacy compared to the clinical anti-CD47 mAbs (141).

Since Fc moiety may have non-specific binding and induce immunogenicity *in vivo*, replacing it with a nanocarrier may improve the application of macrophages-targeted nanobodies. The use of the nanobody-fused nanogels (as a drug nanocarrier) can be an efficient strategy to specifically deplete TAMs by the nanobody moiety and simultaneously deliver a drug surrounded by the nanogel into the TME. For example, the polymeric nanogel-conjugated anti-MMR nanobodies could specifically target MHC-II^low^/MMR^high^ TAMs in the complex environment of tumor cells (136).

The coupling of nanobodies to the other molecules, including small molecules or pro-inflammatory cytokines, can effectively reverse the immunosuppressive microenvironment of tumors following M2-macrophage repolarization. For example, administration of an IFN-γ-/or IL-2-fused high-affinity anti-PD-L1 nanobody to mice bearing pancreatic tumors showed a phenotype shift in intratumoral macrophages toward M1-macrophage with approximately 50% reduction of tumor burden as well as enhanced infiltration of CD8^+^ T cells into TME (142). In another study, a small molecule named imidazoquinoline (IMDQ) was linked to an anti-MMR nanobody. IMDQ have known as a TLR7/8 agonist, which strongly stimulates the inflammatory phenotype of macrophages. *In vivo* studies showed that the use of IMDQ-Linked anti-MMR nanobody resulted in skewed MHC-II^low^MMR^high^ TAMs towards M1-phenotype without its uptake by the other MMR^+^ immune cells such as B cells, dendritic cells (DCs), and monocytes. As a consequence, the anti-tumor function of T cells was augmented, and the tumor burden significantly declined (143).

Although the reprogramming of macrophages can provide a great opportunity for restoring stroma, their effective activation is a major problem for cancer immunotherapy. Simultaneous repolarization of TAMs and blocking of the CD47/SIRPα signaling axis can overwhelm this challenge. It was demonstrated that the cell membrane-coated magnetic nanoparticles engineered to overexpress high-affinity variants of SIRPα mAbs could successfully block the CD47-SIRPα pathway. Moreover, the magnetic nanoparticles promoted M2 TAM repolarization and delivered the cell membrane into tumor sites (144). As a result, the local tumor growth was controlled well, and distant tumor metastasis declined by 50%. The insufficient penetration of nanoparticles into the TME due to the undesirable clearance by the macrophages, as well as their sequestering by monocytes, neutrophils, and RBCs, is a challenge for their translation into the clinic. Nanobodies targeting colony-stimulating factors or CD47 can be suitable substitutes in macrophage-based cancer immunotherapy.

Pre-clinical studies have demonstrated that the reprogramming of macrophages from M2 to M1 phenotype through blocking the surface markers of macrophages such as CCL2, CCL5, PD-L1, as well as blocking the CD47/SIRPa signaling can effectively activate innate and adaptive immune systems to eliminate cancer cells. Moreover, blocking the other vital TAMs receptors (e.g., CSF-1R) and inhibitory receptors (e.g., LILRB1, LILRB2, CTLA-4) by nanobodies can improve the anti-tumor immunity in the TME through reprogramming of macrophages (145, 146). Furthermore, it has been suggested that CAR macrophages can improve anti-tumor functions *in vitro* and *in vivo* models (145). Whether using the nanobody-containing CARs (instead of scFv) can boost the anti-cancer efficiency of the CAR macrophages should be investigated in future studies.

Despite the growing application of nanobodies and increased clinical trials of macrophage-targeted therapies, there is limited literature on targeting macrophages with nanobodies. An anti-MMR nanobody radiolabeled with ^68^Ga-NOTA has entered phase II clinical trials for imaging of MMR-expressing macrophages in head and neck squamous cell carcinoma as well as HL and NHL. The promising results of mAbs that target TAMs for cancer immunotherapy can be expanded to the corresponding nanobodies. A phase I/II clinical study has shown that anti-Clever-1 (bexmarilimab), a novel humanized IgG4-antibody that targets Clever-1 receptor on the TAMs surface, can reprogram M2 TAM to M1 phenotype (147). This antibody has shown considerable anti-tumor activity in patients with advanced solid tumors, including melanoma, gastric, breast, and hepatocellular cancers. Given the desired characteristics of nanobodies, the development of an anti-Clever-1 nanobody could be a suitable candidate for reprogramming TAMs in refractory solid tumors. Moreover, it expects that the identification of new targets on TAMs can increase the application of nanobodies in macrophage-mediated immunotherapy.

## 6 Nanobody-based immune checkpoint Inhibition

**Figure 6:**
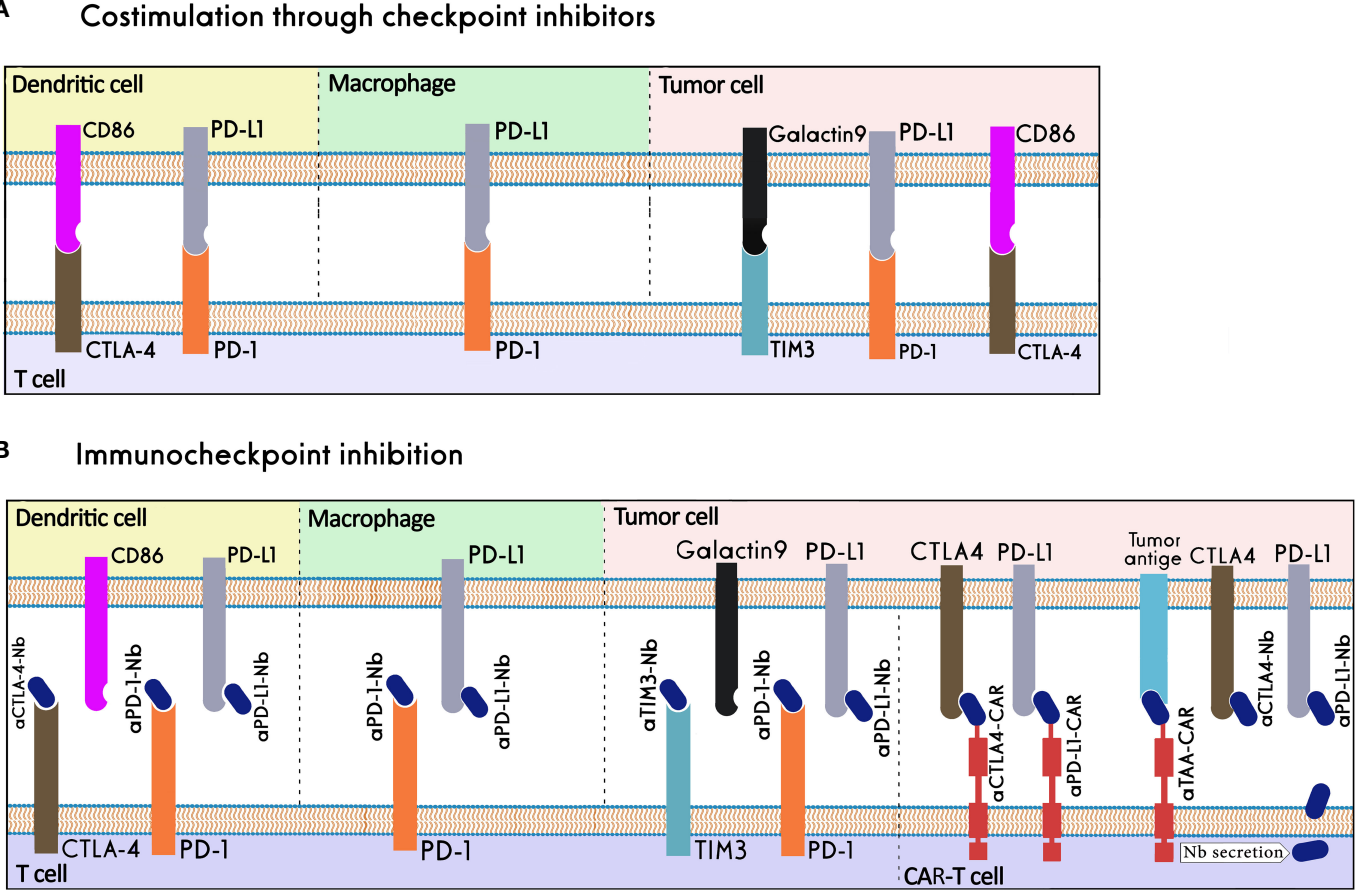
Nanobody-based Immune checkpoint Inhibition. **(A)** Immune checkpoints in physiological conditions. **(B)** Immune checkpoints in the tumor microenvironment, nanobodies can inhibit immune checkpoints by binding to them or their ligands, work as the binding domain in CAR-T cells, or be secreted in the tumor microenvironment by CAR-T cells.

The term "immune checkpoints" (ICs) refers to a group of co-stimulatory and co-inhibitory molecules that are essential for immunological homeostasis and host survival. Under normal physiological circumstances, a balance between the signals from these molecules enables self-tolerance and safeguards the host from tissue damage during an immune response to a foreign antigen (148). In the case of malignancy, immune checkpoint components are co-opted, inhibiting cytotoxic T cells from launching an efficient anti-tumor response (149). Checkpoint inhibitors are medications that stop ICs from binding to their ligands, leading to eliminating T-cell inhibition and boosting the immune system's ability to fight cancer. CTLA-4, programmed death receptor 1 (PD-1), PD-L1, T-cell immunoglobulin and mucin domain-containing 3 (TIM-3), T-cell immunoreceptor with immunoglobulin and immunoreceptor tyrosine-based inhibitory motif (ITIM) domain (TIGIT), and lymphocyte-activation gene 3 (LAG-3) are the receptors that have been the most thoroughly researched (150) (**Figure 6**).

The mAb-based immune checkpoint inhibition has proven to be effective in inhibiting tumor growth. The FDA approved various checkpoint inhibitors as cancer treatments, including mAbs that target PD-1, such as nivolumab (Opdivo®), pembrolizumab (Keytruda®), and cemiplimab (Libtayo®); mAbs against PD-L1, such as atezolizumab (Tecentriq®), avelumab (Bavencio®), and durvalumab (Imfinzi®); and anti-CTLA-4 mAb ipilimumab (Yervoy®). The utility of mAbs has been constrained by factors like high production costs, low tissue penetration, and immune-related side effects (151, 152). The nanobodies' superior properties, compared to mAbs, make them useful tools for successful immune checkpoint inhibition (152).

T, B, and NK cells frequently express the cell surface receptor PD-1. It has been discovered that the PD-1/PD-L1 pathway is important in the escape of cancer from immune surveillance. In many cancers, PD-1 is expressed on effector T cells and exhausted T cells in TME; and PD-L1 is found on the tumor cell surface. One of the most effective strategies to activate anti-tumor immune responses in the tumor environment has been blocking the PD-1/PD-L1 pathway (153). KN035 is a nanobody that can strongly bind to PD-L1 molecules and successfully disrupt the interaction between PD-L1 and PD1. It can successfully contend with other PD-L1 antibodies for the five hotspot areas where PD-L1 binds to PD-1. KN035 can also successfully stimulate peripheral blood mononuclear cells (PBMCs) *in vitro* and trigger interferon release (154). K2, another PD-L1-specific nanobody, could prevent the interaction between PD1 and PD-L1 and improve dendritic cells' capacity to promote T-cell activation and cytokine generation. In contrast to anti-PD-L1 mAbs, this nanobody has a high ability to bind PD-L1 on both immune and non-immune cells and to increase activation of functional antigen-specific cytotoxic T cells. Therefore, treatment with K2 nanobody and dendritic cell vaccination may be more effective against cancer diseases than PD-L1 mAbs (155). Moreover, an anti-PD1 nanobody, Nb97, was used to create the Nb97-Nb97-HSA fusion protein that demonstrated more efficacy in stimulating the immune function than a humanized Nb97-Fc (156). In order to draw the appropriate leukocyte populations in the TME, a PD-L1-blocking nanobody was fused to a charge-engineered chemokine CCL21. Using a microfluidic platform mimicking the TME, it was demonstrated the chemokine-nanobody fusion could selectively target PD-L1^+^ tumor cells and recruit effector cells to the TME (157). A unique anti-PD-L1/CXCR4 bispecific nanobody could successfully penetrate the tumor tissues in a xenograft mouse model and inhibit the growth of pancreatic cancer cells much better than the combination therapy using anti-PD-L1 and anti-CXCR4 nanobodies (158).

A bispecific antibody consisting of an anti-PD-L1 antibody and an anti-TIGIT nanobody could bridge between PD-L1^+^ tumor cells and T/NK cells leading to simultaneous blockade of PD-L1/PD-1 and TIGIT, enhanced infiltration of cytotoxic T lymphocytes (CTLs) and NK cells; and tumor growth inhibition in a mouse model (159). Moreover, a PD-L1/4-1BB bispecific nanobody can concurrently bind PD-L1 on tumor target cells and 4-1BB on effector cells. It can block the PD-L1/PD-1 pathway and induce 4-1BB signaling at the same time, leading to high antitumor potency with minimal toxicity (56).

A key inhibitory regulator of immunological responses is CTLA-4. Following T-cell activation, CTLA-4 rapidly expresses on T cells and has a higher affinity than CD28 for binding to B7 molecules. By increasing the threshold of signals needed for full T-cell activation, CTLA-4 may make it impossible for T cells to begin responding, and it may also cause them to stop responding already (148). Using phage display, four CTLA-4-specific nanobodies were isolated that had a strong binding capacity and were able to detect particular CTLA-4 epitopes. The survival of mice with melanoma increased after receiving the anti-CTLA-4 nanobody (Nb16) treatment because of the significant tumor growth inhibition (160). Moreover, this anti-CTLA-4 nanobody could increase the anti-tumor activities of cytotoxic T cells and prolong the survival of NOD/SCID mice bearing melanoma cells (151). The immunosuppression caused by the CTLA-4 pathway may limit the effectiveness of cellular therapy based on the tumor antigen-presenting dendritic cells and cytokine-induced killer cells (DC-CIK). An anti-CTLA-4 nanobody was used to suppress CTLA-4 signaling in order to counteract the negative co-stimulation from T cells. The DC-CIK cells displayed increased proliferation and IFN-γ production *in vitro* following stimulation with anti-CTLA-4, which intensified their lethal effect on the tumor cells (161). Another strategy to prevent ICs is to give nanobodies locally using CAR-T cells as cargo. The CAR-T cells that secrete anti-PD-L1 or anti-CTLA-4 nanobodies have better proliferation and persistence compared with nonsecreting CAR-T cells (162).

Alternative ICs, including TIM3, can be considered as a potential therapeutic target to obtain more effective anti-tumor responses in patients not responding to PD1/PDL1 and CTLA4 inhibitors (163). An anti-human TIM-3 nanobody was produced with an inhibitory impact equivalent to or superior to that of anti-TIM-3 antibodies. This nanobody demonstrated a high binding capacity to TIM-3 and a high anti-proliferative effect on the acute myeloid leukemia cell line HL-60 by disrupting the galectin/TIM-3 signal (164).

Considering their excellent tissue penetration properties, nanobodies are attractive candidates for blocking inhibitory receptors of immune cells in the dense environment of solid tumor tissues. A nanobody targeting CTLA-4 has entered a phase I clinical trial for treating advanced/metastatic solid tumors (NCT04126590). In addition, anti-mesothelin CAR-T cells secreting PD-1 nanobodies are under clinical evaluation for mesothelioma, ovarian, colorectal, and non-small-cell lung cancers (NCT04489862, NCT05373147, NCT04503980).

## 7 The potential role of nanobodies in managing adverse effects of immune cell therapy

**Figure 7:**
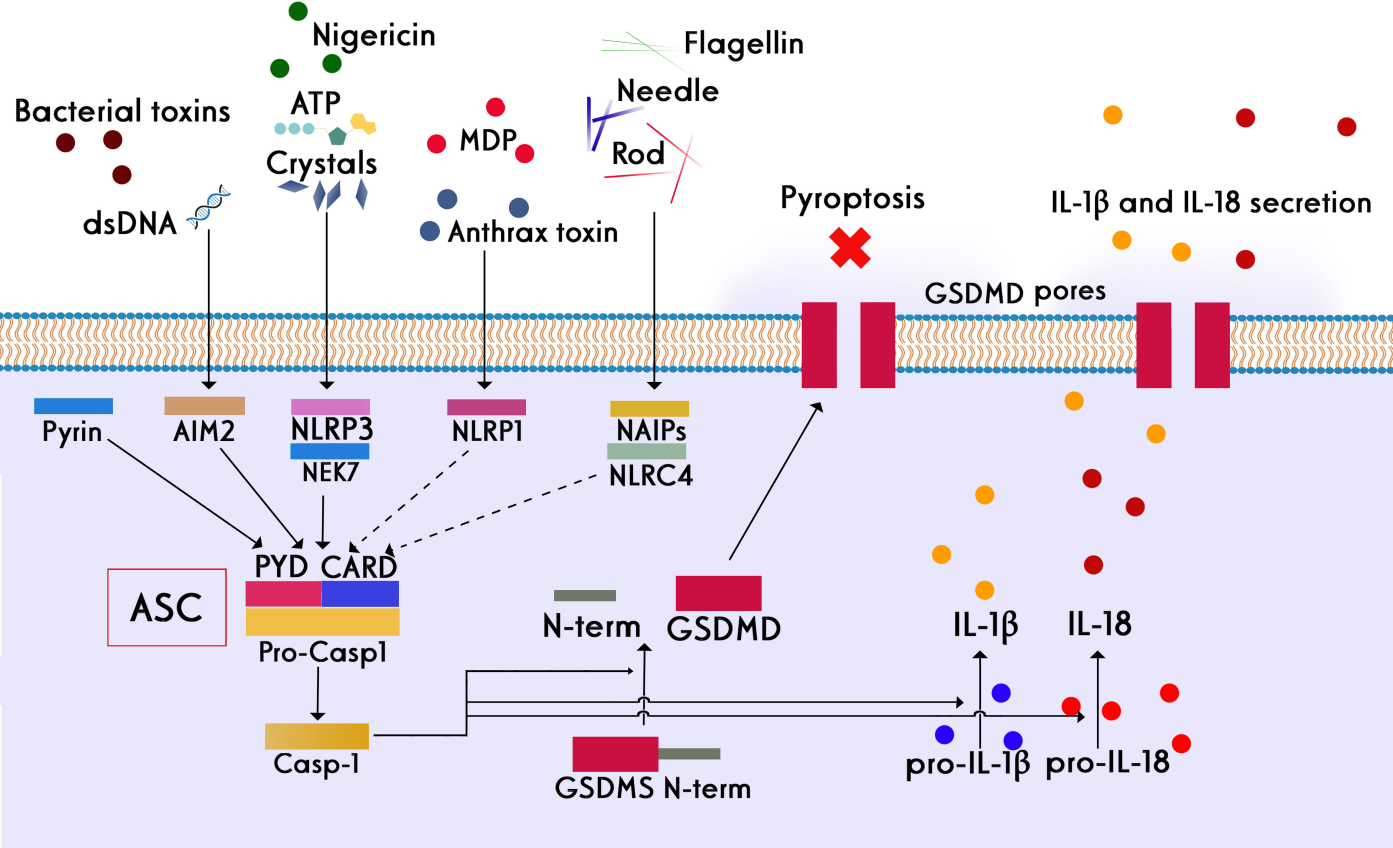
Different stimuli involved in the activation of different subsets of the inflammasome family and the canonical role of ASC in inflammasome assembly. Four key inflammasomes, namely NLRP1, NLRP3, NLRC4, and AIM2, have been best characterized. Following the activation of inflammasomes, inflammatory caspases are converted from the pro-active to the active form. The main consequence of this event is the creation of the active form of the pro-inflammatory cytokine interleukin 1 beta, which is one of the main drivers of inflammation. Apoptosis inhibitory proteins (NAIPs) function as direct receptors for bacterial flagellin and the needle and rod subunits. NLRP3 activation also requires NIMA-related kinase 7 (NEK7), which binds to the NLRP3 leucine-rich repeats (LRRs) and is required for its oligomerization. Many stimuli, such as bacterial lipopolysaccharide (LPS), crystals, extracellular ATP, and dsDNA, can activate the inflammasome complex. In addition to creating the active form of gasdermins, the consequence of inflammasome activation is the secretion of significant amounts of pro-inflammatory cytokines, such as IL-1β and IL-6, by macrophages, which are the main drivers of inflammatory events during CRS.

Despite the clinical success of BiTEs and CAR-T cells in the treatment of B-cell malignancies, CRS and its subsequent inflammatory effects reduce the effectiveness of these therapies in patients (165). Pyroptosis is an inflammatory cell death that is caused by pore-forming proteins such as Gasdermins (E and D). These proteins are activated by inflammatory caspases and cause pores in the cell membrane that results in releasing their cytosolic content containing high amounts of danger-associated molecular patterns (DAMPs). The DAMPs contribute to the initiation of the inflammasome activation and subsequent activation of macrophages, which produce high levels of proinflammatory cytokines such as IL-1β and IL-6, the main drivers of inflammatory events during CRS (166, 167). All types of inflammasomes require an adapter protein called ASC (apoptosis-associated speck-like protein containing a CARD, caspase recruitment domain) to be activated (**Figure 7**) which includes a pyrin domain (PYD) and a CARD. The main feature of inflammasome activation is the formation of ASC speck as a result of the oligomerization of ASC (PYD) filaments and their cross-linking with CARD, which creates a micrometer-sized structure (168). The blockage in these pathways can lead to disruption in inflammation.

A nanobody was developed against the CARD domain of human ASC protein to investigate how the inflammasome is activated under physiological conditions. *In vitro* studies have shown that this nanobody not only prevents the interaction between CARD and PYD but also inhibits the activation of various types of inflammasomes which indicates the importance of ASC protein in the construction of different types of inflammasomes (169). It was proved that this nanobody was able to inhibit the inflammatory function of post-pyroptotic inflammasomes, making nanobodies the first biological agents capable of disassembling precursors of inflammasomes *in vitro*. Nanobodies against human and mouse ASC could effectively inhibit ASC-Speck activity after pore formation in pyroptotic cells, whereas they did not interfere with the initial secretion of cytokine IL-1β before pyroptosis, which is necessary for host defense against infections (170).

P2X7 is a ligand-gated ion channel that detects extracellular ATP and initiates a pro-inflammatory signaling cascade that results in NLRP3 inflammasome activation and secretion of pro-inflammatory cytokines. The P2X7 ion channel is prominently expressed by monocytes and T cells and responds to extracellular ATP from damaged cells during sterile inflammation. Specific small-molecule inhibitors for P2X7 were developed for the treatment of inflammatory diseases by Pfizer (CE-224,535) and Astrazeneca (AZ-10606120 and AZD9056) (171, 172).

Studies show limitations of small molecule inhibitors, including short half-life *in vivo*, narrow therapeutic window, or toxic metabolites (173). Due to the unique propensity of nanobodies to bind to functional crevices on proteins, these molecules can resolve the need for special bindings to ion channels (174). Highly potent nanobodies were isolated against mouse and human P2X7 ion channels, which showed higher specificity and lower toxicity in comparison to small molecule antagonists. The anti-mouse P2X7 nanobody could efficiently decrease inflammation in an experimental mouse model of glomerulonephritis. Compared with small-molecule inhibitors of P2X7, the anti-human P2X7 nanobody showed much more potency in preventing the IL-1β release, making it a viable biological option for controlling inflammatory cases associated with high content of cell damage and extracellular ATP (175). Another important player involved in the pathogenesis of CRS and neurotoxicity effects induced by CAR-T treatment is the pro-inflammatory cytokine IL-6. Inhibition of IL-6 signaling by tocilizumab, a mAb against IL-6 receptor (IL-6R), has been approved by the FDA as a biological treatment for the CAR-T-related CRS (176).

Unlike other cytokines, IL-6 can activate the signaling cascade by binding to a membrane receptor (mIL-6R; classical signaling) or to its soluble receptor (sIL-6R; trans-signaling) (177). A half-life extended bispecific nanobody was developed against IL-6R and HSA (human serum albumin) under the name ALX-0061. Different studies on this molecule proved its ability to inhibit IL-6 signaling equivalent to the tocilizumab antibody. ALX-0061 nanobody showed increased half-life and complete inhibition of inflammation in a monkey model of collagen-induced arthritis. Considering the promising results of this molecule in preclinical experiments, it is expected to be used as a biotherapeutic in the control of chronic inflammation and autoimmune diseases (178).

## 8 Conclusion

Antibodies are being widely explored for the activation of immune cells, including T cells, NK cells, and macrophages. Moreover, the retargeting of immune cells toward cancer cells has been achieved through tumor antigen-specific antibodies. Interestingly, antibodies could reverse the immunosuppressive state of the TME by immune checkpoint blockade. The superior properties of nanobodies compared to conventional antibodies make them ideal candidates for targeted modulation of immune cells in cancer therapies. Thanks to their high stability, enhanced infiltration into the TME of solid tumors and facile genetic engineering, nanobodies can act as complements to immune cell-mediated therapies. However, the fast clearance of nanobodies from circulation may preclude their utility for therapeutic applications. To improve their *in vivo* half-life, nanobodies can be modified by different approaches such as PEGylation, PASylation, addition of albumin-binding domains, and fusion to Fc domain of human antibodies.

The role of nanobodies in cell-mediated immunotherapy can be categorized into three types: bridging between tumor and immune cells, targeting tumor cells with immune cell-bound nanobodies, and blocking the inhibitory receptors expressed on immune cells (**Figure 1**). The nanobody-based bi- and multispecific cell engagers can make a bridge between tumor cells and immune effector cells leading to cell-mediated cytotoxicity. As T and NK cells’ engagers, nanobodies have shown promising preclinical results in multiple myeloma and breast cancer, which provide insights to develop more specific nanobodies against the surface antigens of cancer and immune cells. The most successful application of nanobodies in cell-mediated immunity has been redirecting immune cells to attack tumor cells through a chimeric receptor incorporating a nanobody against the target antigen. Various cancer antigens have been targeted by nanobody-based CAR-T and CAR-NK cells for treating both hematological and solid malignancies. However, considering their clinical trials results, anti-BCMA NbCAR-T cells may be the first nanobody-based engineered immune cells to reach the market for treating multiple myeloma. Finally, by blocking the immune cells’ inhibitory receptors, nanobodies can aid them to exert more efficient anti-tumor functions. As the potent immune checkpoint inhibitors, nanobodies can reverse the T cell exhaustion and restore anti-tumor immunity. Moreover, the anti-tumor functions of macrophages can be enhanced through nanobody-mediated blockade of the CD47/SIRPa pathway, reprogramming of macrophages from M2 to M1 phenotype and depletion of TAMs from the TME. Nanobodies against the inflammatory cytokines and proteins involved in pyroptosis can be used to prevent the severe side effects occurring in CAR-T cell therapy and might enable a safer anti-cancer immune cell therapy.

Taken together, nanobodies are appealing as potential therapeutics to effectively modulate the immune cells’ functions, manage the inflammatory side effects of immune cell therapy and restore the immune surveillance to cancer patients.

## Author contributions

ZS supervised and revised this manuscript. AM, MG, SF-N, AN, SS, and SM contributed to data collection, literature review, and manuscript drafting. All authors contributed to the article and approved the submitted version.
